# Review of Evaluation Metrics Used in Digital and Traditional Tobacco Control Campaigns

**DOI:** 10.2196/17432

**Published:** 2020-08-11

**Authors:** Lilian Chan, Blythe O'Hara, Philayrath Phongsavan, Adrian Bauman, Becky Freeman

**Affiliations:** 1 Sydney School of Public Health and Charles Perkins Centre Prevention Research Collaboration The University of Sydney Camperdown Australia

**Keywords:** mass media, internet, evaluation studies as topic, smoking cessation, public health

## Abstract

**Background:**

Mass media campaigns for public health are increasingly using digital media platforms, such as web-based advertising and social media; however, there is a lack of evidence on how to best use these digital platforms for public health campaigns. To generate this evidence, appropriate campaign evaluations are needed, but with the proliferation of digital media–related metrics, there is no clear consensus on which evaluation metrics should be used. Public health campaigns are diverse in nature, so to facilitate analysis, this review has selected tobacco control campaigns as the scope of the study.

**Objective:**

This literature review aimed to examine how tobacco control campaigns that use traditional and digital media platforms have been evaluated.

**Methods:**

Medicine and science databases (Medical Literature Analysis and Retrieval System Online [MEDLINE], EMBASE, PsycINFO, Cumulative Index to Nursing and Allied Health Literature [CINAHL], and Scopus), and a marketing case study database (World Advertising Research Center) were searched for articles published between 2013 and 2018. Two authors established the eligibility criteria and reviewed articles for inclusion. Individual campaigns were identified from the articles, and information on campaigns and their evaluations were supplemented with searches on Google, Google Scholar, and social media platforms. Data about campaign evaluations were tabulated and mapped to a conceptual framework.

**Results:**

In total, 17 campaigns were included in this review, with evaluations reported on by 51 articles, 17 marketing reports, and 4 grey literature reports. Most campaigns were from English-speaking countries, with behavioral change as the primary objective. In the process evaluations, a wide range of metrics were used to assess the reach of digital campaign activities, making comparison between campaigns difficult. Every campaign in the review, except one, reported some type of engagement impact measure, with website visits being the most commonly reported metric (11 of the 17 campaigns). Other commonly reported evaluation measures identified in this review include engagement on social media, changes in attitudes, and number of people contacting smoking cessation services. Of note, only 7 of the 17 campaigns attempted to measure media platform attribution, for example, by asking participants where they recalled seeing the campaign or using unique website tracking codes for ads on different media platforms.

**Conclusions:**

One of the key findings of this review is the numerous and diverse range of measures and metrics used in tobacco control campaign evaluations. To address this issue, we propose principles to guide the selection of digital media–related metrics for campaign evaluations, and also outline a conceptual framework to provide a coherent organization to the diverse range of metrics. Future research is needed to specifically investigate whether engagement metrics are associated with desired campaign outcomes, to determine whether reporting of engagement metrics is meaningful in campaign evaluations.

## Introduction

### Background

By 2019, advertising on the internet made up over half of all media spending in 8 countries, including the United Kingdom, China, the United States, and Australia [1]. The growing trend toward digital advertising has extended into public health mass media campaigns, with the majority of these campaigns now using digital media platforms, such as web advertising and social media, in addition to traditional media platforms [2].

Despite the increasing popularity of digital media use, there is a lack of robust evidence on how best to use digital platforms for public health campaigns, including questions around which platforms, or combinations of platforms, are most effective for driving behavioral change [3]. Developing a body of evidence in this area is vital to ensure public health campaigns are effective, that they reach intended audiences, and that there is appropriate investment of resources.

To generate this evidence, appropriate evaluations of campaigns are needed. With the proliferation of digital media platforms, metrics such as likes, engagements, impressions, and click-through rates have become commonplace in evaluations [3-8]. Despite the prevalence of their use, their meaning in public health is not completely understood, and there are currently no clear guidelines on which, if any, of these metrics are relevant for public health campaign evaluations. This situation will continue to become a greater challenge, as the continual emergence of new platforms, such as the recent popularity of Tik Tok (ByteDance) [9], leads to an ever-increasing number of digital evaluation metrics. In addition, the growing number of digital media platforms means that campaigns can use multiple media platforms, creating the additional challenge for practitioners to understand which platform, or combination of platforms, should be used for public health campaigns.

Given varied objectives, strategies, and activities of public health campaigns, this review focuses on campaigns relating only to tobacco control to facilitate comparison. Today, some tobacco control campaigns are among the most advanced public health campaigns in terms of funding, strategy, and evaluation, and have a large underpinning evidence base that describes effective campaigns [10]. Despite this, there is limited evidence on what constitutes effective digital media use in tobacco control campaigns, with the US Center for Disease Control and Prevention’s Best Practices for Comprehensive Tobacco Control Programs acknowledging that there is insufficient evidence to make any recommendations on how to best use digital media channels [11]. This gap in knowledge is the background for this review.

### Objectives

This paper examines how tobacco control campaigns that use traditional and digital media platforms have been evaluated in the published literature. A better understanding of how to evaluate these campaigns will enable practitioners and researchers to develop greater insight into how to effectively use digital media platforms for tobacco control campaigns, and more widely, for public health campaigns.

## Methods

### Data Collection

Data were collected through 3 search approaches: (1) in medicine and science journal databases, (2) in a marketing case studies database, and (3) through internet searches for grey literature, campaign websites, and social media sites.

For medicine and science journals, a search was conducted using the Medical Literature Analysis and Retrieval System Online (MEDLINE) via OvidSP (Wolters Kluwer Health), EMBASE via OvidSP, PsycINFO via OvidSP, and Cumulative Index to Nursing and Allied Health Literature (CINAHL; EBSCO) and Scopus (Elsevier). The search strategy used the following terms: (smok*.mp OR tobacco/) AND (campaign.mp OR mass media.mp) AND (digital.mp OR online.mp). Search results were limited to articles in English and published in the last 5 years (2013-2018). This timeframe was selected to ensure the relevance of this review because of the fast-changing nature of digital platforms and their usage patterns.

The review was supplemented with a search of the marketing database WARC (World Advertising Research Center). For this search, the keyword terms were smoking OR tobacco, with results limited to the last 5 years, within the *Non-profit, public sector, and education* database category.

Subsequently, the reference lists of included articles and systematic reviews identified in the literature search were reviewed for additional relevant references.

The first stage of this review involved 2 authors (LC and BH) independently reviewing the same subset (25%) of all identified database search results to establish and test the eligibility criteria (see Multimedia Appendix 1)*.* One author (LC) then reviewed the remaining search results against the criteria to identify literature that warranted full-text review. The same 2 authors then independently reviewed all full-text articles against the eligibility criteria.

### Campaign Identification

**Figure 1:**
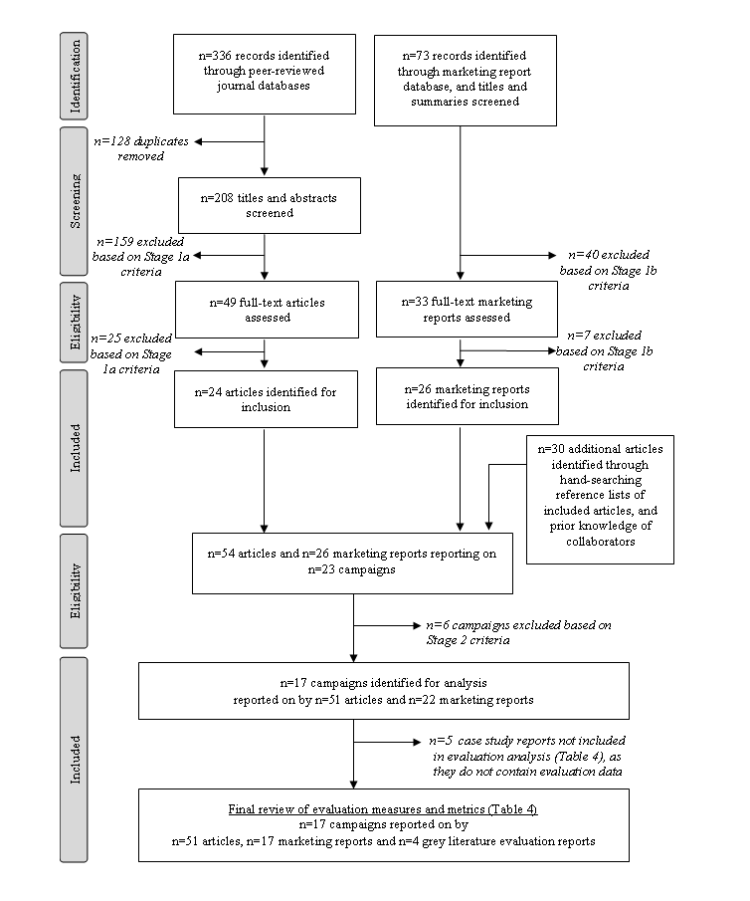
Flowchart of search strategy and campaign selection.

The second stage of the literature review involved the identification of individual campaigns from the included articles (see Figure 1). Each identified campaign was searched on both Google Scholar and Google for evaluation reports, press releases, or other evaluation materials. Campaign websites and social media pages were also searched and examined. Based on these multiple sources, campaigns were assessed for inclusion in the review against the eligibility criteria (see Multimedia Appendix 1). One author (LC) conducted the additional searches and performed the initial assessment against the eligibility criteria. Two authors (BH and BF) independently reviewed any unresolved campaigns.

### Data Extraction

All articles identified throughout the data collection process were recorded using Endnote (Version X8, Clarivate Analytics). Information from multiple sources was then tabulated by campaign to provide a complete picture of the evaluation measures and methods used by each campaign. To provide context for the evaluations, data on each campaign’s objectives, target audience, and details of media usage (both paid and unpaid) were also collected.

### Data Analysis

To summarize evaluation measures used by different campaigns, data were mapped to a conceptual framework (Table 1). This framework includes evaluation metrics that were commonly reported for the digital components of campaigns, alongside measures that have conventionally been used in campaign evaluations [12,13]. The conceptual framework is based on the different levels of evaluation—process, impact, and outcome. Building on other campaign evaluation models [12,13], this framework incorporates several levels of impact evaluation: measures of campaign awareness, engagement, priming steps, and trialing behaviors (Table 1). Actions within each level of evaluation are not necessarily equal in value to the overall campaign outcome but are grouped together based the nature of the action. Information on whether and how campaigns measured which media platforms contributed to outcomes was also collected. Formative, precampaign, and message development evaluations were not included in this review.

**Table 1:** Conceptual framework of campaign evaluation metrics and measures

Process evaluation	Impact evaluation				Outcome evaluation
	Awareness	Proximal impact I: Engagement	Proximal impact II: Priming steps	Distal impact: Trialing behaviors	
					
*Delivery of campaign*	Seen the campaign and perception of the campaign	Showing interest in the campaign or message by taking an action	Priming steps of behavioral change	Initial trialing behaviors and antecedents of behaviors	Desired behavioral change
Delivery of Television ads (Target Audience Rating Points [TARPs] or Gross Rating Points [GRPs]) *Digital video ads (digital GRPs or impressions or video views)*^a^ Digital banner ads (impressions or exposures) Other	Campaign recall (including frequency) *Media channel attribution (where campaign was viewed)b* *Campaign response (eg, relevance, perceived effectiveness, believability)b*	*Campaign website visits* *Engagement on social media (eg, likes, comments, shares, follows)* *Click through rates (on digital ads or social media posts)* *Information-seeking action on the internet (web search)* *Other action (eg, download mobile app, sign up to campaign)*	Knowledge and beliefs Attitudes: about smoking, tobacco industry, etc Attitude: intention to quit Information-seeking action offline (spoke with health care provider)	Contact smoking cessation service or registrations to service Quit attempts	Sustained quit attempts Population smoking prevalence rates (For nonsmokers): Conversation with family or friend about smoking cessation

^a^All italics indicate metrics and measures that relate to digital media platforms.

^b^In this review, media channel attribution and campaign responses were measured through both digital platform evaluation methods and traditional evaluation methods.

## Results

### Study Selection

The medicine or science database searches identified 336 articles. After removal of duplicates, 208 articles were screened. This identified 49 articles for full-text review, and subsequently 24 articles were included in this review. The marketing database search identified 73 reports, and after review, 26 were included. From hand-searching references of the included articles, 30 additional articles were identified for this review (see Figure 1).

### Campaign Selection

After further searches for more information about the identified campaigns in grey literature reports, campaign websites and social media pages, 6 campaigns were excluded for the following reasons: insufficient information about the campaign, insufficient information about the digital aspects of the campaign, lack of evaluation data, campaign related to e-cigarettes, and intervention assessed as not primarily a campaign. As a result, 17 campaigns were included in this review, reported on by 51 peer-reviewed articles and 22 marketing reports. However, 5 of the marketing reports provided contextual campaign information but did not contain unique evaluation data. Therefore, the analysis of evaluations of the 17 campaigns was based on 51 peer-reviewed articles, 17 marketing reports, and 4 grey literature evaluation reports.

Of the 17 identified campaigns, 7 were only located in marketing reports and grey literature, highlighting the benefit of using these additional sources of information for this review. Of the 51 peer-reviewed articles included in this review, 29 reported on the *Tips from Former Smokers* campaign, 7 reported on the *Truth FinishIt* campaign, and 7 reported on *The Real Cost* campaign.

### Campaign Characteristics

Most campaigns were from high-income, English-speaking countries, with 6 from the United States, 4 from Canada, 3 from Australia, and 2 from the United Kingdom. In all, 13 of the 17 campaigns had a primary objective of behavioral change, 2 were awareness-raising campaigns, and 2 were campaigns aimed at changing social norms.

### Campaign Evaluation Measures

The types of evaluation measures used for campaigns are summarized in Tables 2 and 3.

**Table 2:** Reported evaluation measures in behavioral change campaigns.

Campaign	Process	Awareness	Proximal impact: engagement	Proximal impact: priming steps	Distal impact	Outcome
Tips from Former Smokers	✓	✓	✓	✓	✓	✓
Stop before the suffering starts	✓	✓	✓	✓	✓	—^a^
Stoptober	—	✓	✓	✓	✓	✓
The Real Cost	✓	✓	✓	✓	—	✓
Be a Failure	✓	✓	✓	✓	—	—
16 cancers	—	✓	✓	—	✓	—
SmokeFree Teen	✓	—	✓	—	✓	—
Fingerband campaign	✓	✓	✓	—	—	✓
Break it Off	—	—	✓	✓	✓	✓
Keep Trying	—	—	✓	—	✓	—
No judgments. Just help	—	—	✓	—	✓	✓
Personal Testimonies	—	—	✓	—	✓	—
The Smoking Kid	✓	—	—	—	✓	—

^a^No data was available on these evaluation measures.

**Table 3:** Reported evaluation measures in awareness raising and social norm change campaigns.

Campaign	Process	Awareness	Proximal impact: engagement	Proximal impact: priming steps	Distal impact or outcomes
Truth FinishIt	✓	✓	✓	✓	✓
The Facts Now	✓	—^a^	✓	—	✓
Take it right outside	—	✓	✓	✓	✓
Quit the Denial	✓	✓	✓	✓	—

^a^No data was available on these evaluation measures.

#### Process Evaluation Measures

The conceptual framework as described in Table 1 emphasizes quantitative measures for process evaluations of campaigns. Of the 10 campaigns in this review that had a television advertising component, 4 reported the number of target audience rating points (TARPs) or gross rating points (GRPs) [14-23], which are both measures of reach, describing the estimated percentage of the population that viewed the ad.

The majority of campaigns (8/10) using digital videos reported a metric about the reach of the digital video [8,15,19,24-32]. The reach of digital videos was reported using a variety of metrics, including digital TARPs (the equivalent of TARPs for content delivered on a digital platform) [33], impressions (the number of times the content was delivered) [33], exposures (opportunities for the content to be seen [34]), or video views.

The reach of web banner ads was reported as impressions or exposures by 2 campaigns [8,24], and digital impressions by 1 campaign, but it was not clear whether this was for static banner ads and/or digital video ads (*Truth FinishIt*) [35]. One campaign reported measuring banner ad reach but did not report the result (*Be a Failure*) [36].

#### Campaign Awareness Measures

In all, 7 campaigns evaluated whether people recalled (ie, without prompting with campaign material) or recognized (after being shown campaign material) the campaign, which was primarily measured through sampled surveys or interviews [14,15,19,23,26,35,37-54]. A total of 7 campaigns reported evaluations on the audience’s response to the campaign, such as perceived effectiveness of the campaign or emotional reaction to the campaign. This was evaluated through surveys or interviews or content analysis of social media comments [14,15,25,30,36,51,53-58].

#### Proximal Impact Evaluation Measures I: Engagement

Proximal impact measures of engagement, such as the number of visits to a website or ad click-through rates (the percentage of times an ad is clicked) [33], represent intermediary steps between exposure to a campaign and the desired outcomes of a campaign (see Table 1).

All but one campaign in this review reported at least one proximal impact measure of engagement. Of all the evaluation measures identified in this review, campaign website visits was the most commonly reported measure (11/17 campaigns) [8,20,24,27,36,37,59-66]. Engagement on social media—broadly encompassing numbers of likes, shares, comments, or followers on any social media platform—was reported for 8 campaigns [8,25-27,29,30,32,35,52,60]. Two of these campaigns used aggregated metrics of engagement (social media engagement rate in *The Real Cost*, and social media conversation in *Quit the Denial*) [26,29].

The number of times an ad was clicked or the click-through rate were only reported in 2 of the 11 campaigns that used web static banner ads (*SmokeFree Teen* and *Tips from Former Smokers*) [8,24].

In all, 5 campaigns reported on whether people exposed to the campaign took an intermediary action of seeking more information about the issue on the internet [14,24,26,36,67,68]. This was either measured through survey questions or through analyzing campaign keyword search trends on search engines (*Tips from Former Smokers* and *Stoptober*) [67,68].

A total of 5 campaigns used other digital media–based measures as part of the evaluation of proximal impact. These included measuring mobile phone app downloads [8,14,60,63], sign ups to the campaign [32], views of email marketing messages [69], and campaign resource downloads [63].

#### Proximal Impact Evaluation Measures II: Priming Steps

In all, 3 of the 17 campaigns measured knowledge-related outcomes, such as about the health-related harms of smoking or of second-hand smoke [26,40,46,50,70]. A total of 8 campaigns measured attitudes related to smoking, the tobacco industry, and the quitting process [14,23,26,36,39,40,42,43,45,46,51-54,64,67,70,71]. Overall, 8 campaigns specifically measured attitudes around intention to quit smoking [14,21,26,36,39,45,47,48,50,53,60,63]. Changes in knowledge and attitudes were measured by surveys or interviews. In addition, 3 campaigns identified whether people had spoken to a health care professional for more information on quitting [14,26,36].

#### Distal Impact Evaluation Measures: Trialing Behaviors

The number of people contacting smoking cessation services was reported in 9 of the 13 behavioral change campaigns [8,14,18,22,28,37,59,61,65-67,72,73]. In all, 6 campaigns evaluated the number of people making quit attempts [14,17,21,40,44,46,47,50,60,63,67,72,74,75].

#### Outcome Evaluation Measures

Finally, 4 campaigns evaluated the number of people with sustained quit attempts [44,47,60,63,72,76]. *The Real Cost,* which aimed to reduce smoking initiation rates in young people, evaluated smoking initiation behavior [41]. *Tips from Former Smokers,* which had nonsmokers as a secondary target audience, also measured the number of nonsmokers who had initiated conversations about smoking cessation with friends or family [44,50]. These outcomes were all measured by surveys or interviews. In addition, 2 campaigns (*Fingerband Campaign* and *The Facts Now*) used population smoking prevalence rates [25,27], and 1 campaign (*Stoptober)* measured cigarette sale volumes as part of the outcome evaluation [67].

#### Media Platform Attribution

In all, 7 campaigns attempted to measure media platform attribution, that is, where the audience was exposed to the campaign [8,14,19,35,37,38,40,44,59]. A total of 4 campaigns used surveys or interviews to ask participants where they recalled seeing the campaign (*Stop before the* s*uffering starts, Tips from Former Smokers, Take it right outside,* and *Truth FinishIt*) [14,19,35,40,44], 2 campaigns used correlations between timings of campaign outcome events with waves of the campaign that used different media formats (*16 Cancers* and *Personal Testimonies*) [37,59], and 1 campaign used unique website tracking codes for ads shown on different media formats (*SmokeFree Teen*) [8].

## Discussion

### So Many Metrics, Which Ones to Use?

This review found that there is a wide range of metrics used in tobacco control campaign evaluations, as a consequence of the diversity of media platforms and activities employed by campaigns (see Multimedia Appendix 2 [5,8,15-32,35-63,65-85]). While this gives the impression that there is a lot of information about how a campaign performed, in reality the large number of metrics makes it difficult to meaningfully interpret the reported numbers. For process evaluations, there was a gap between evaluations of traditional media use, such as television ads which used the standardized metrics of GRPs or TARPs, compared with digital media platforms which used a variety of metrics including reach, impressions, exposures, video views, and digital GRPs. The diversity in metrics is partially because of the fragmented media landscape, with each digital media platform having its own reporting system. As all the metrics refer to slightly different measures, it makes comparisons between campaigns difficult. In addition, these raw reach metrics on social media may not reflect a broad generalized reach, as one of the criticisms of organic social media activity is that it perpetuates echo chambers, where messages are often only shared between like-minded individuals. This is less of an issue when campaigns use paid social media strategies, where they can choose the target audience of the campaign ads based on demographics, stated interests, and previous online behavior.

Another group of metrics identified in this review were engagement metrics, which result from digital media activities, and were not present in traditional broadcast media. Examples of these metrics included likes, comments, and retweets. The sheer number of these engagement metrics is overwhelming, and it is challenging to know which are meaningful [86,87]. An additional type of metric identified in this category are metrics which are amalgamations of other metrics, such as social media engagement and social conversation. These have usually been created by advertising companies, and the calculation of these metrics is usually not transparently described. Finally, digital metrics are usually provided by the platforms themselves, which raises a number of issues. First, the platforms are constantly changing their reporting systems. For example, in 2019 Facebook and Instagram began hiding the number of likes publicly displayed [88,89]. Second, the metrics are not open to independent scrutiny as the platforms are not transparent in how the metrics are calculated. For example, Facebook has previously been reported to have inflated its video view metrics [90]. With these factors in play, campaign practitioners are faced with the great challenge of deciding which metrics to use.

There are currently moves to try to create more uniform digital metrics across the board [91-93]; however, this is a complex undertaking and it is unlikely that a standardized system will be developed in the near future. In the meantime, a published glossary explaining commonly used metrics could provide practitioners and evaluators with a greater understanding of the specific definitions of metrics. In addition, when practitioners and evaluators select metrics, they should be guided by certain principles, as opposed to overloading the reader with numbers that may or may not have relevance to the evaluation. Principles to guide the use of metrics include the following:

1. Metrics should be consistent with the objectives of the campaign [87,94]. For example, reach (the number of people who have seen a campaign) would be appropriate for awareness-raising campaigns that aim to reach as many people as possible, whereas impressions (the number of times the campaign has been shown to the target audience) could be more relevant for behavioral change campaigns that aim to communicate a message many times to a targeted audience.
2. Reported metrics should be the simplest metric available for reporting the intended concept, that is, the metric understood by most people. While complex metrics may help practitioners understand how campaigns are performing at the time, they are usually not widely understood. Furthermore, combined metrics, such as “the campaign produced XXX impressions in total,” should be avoided, as they are ambiguous about how the number is calculated across different media.

### Contextualizing Evaluation Metrics Through the Conceptual Framework

The conceptual framework in Table 1 provides a starting point in organizing the range of metrics identified in this review. The framework is based on an established program evaluation framework, and for the purposes of planning and evaluating campaigns, provides a structured approach to grouping the metrics. In reality, the flow of events relating to the campaign-desired outcomes may not be linear as depicted in this framework. In the public health literature, several approaches have been used to organize social media metrics [93,95-97]; however, they focus on social media metrics alone, without demonstrating how the social media metrics fit with other digital media measures or other mass media evaluation measures.

Through the use of this conceptual framework to review the range of metrics, we identified strengths and gaps in the evaluations in this review. A large proportion of campaigns reported proximal impact engagement measures, such as website visits, whereas a smaller proportion evaluated proximal impact priming step measures of health-related knowledge and attitudes. The review also identified that marketing reports generally focused more on process evaluation measures and proximal impact engagement measures, whereas peer-reviewed articles focused more on priming step measures. This distinction has practical implications, as campaigns with smaller evaluation budgets often rely on marketing reports to evaluate the effectiveness of a campaign. Conversely, researchers may only look at peer-reviewed articles to identify best practice in campaign development. As all levels of evaluation are of value, it is important that the full spectrum of evaluation measures is reported to understand the effectiveness of a campaign.

Many mass media campaigns are based on behavioral change theories that have priming steps of changes in knowledge, attitudes, or beliefs as intermediary stages before the behavioral change outcome [15,98]. This conceptual framework demonstrates that there is a gap in understanding of whether there is any relationship between proximal impact engagement measures (such as Facebook likes) and proximal impact priming steps of changes, or other impact or outcome measures. Social media is inherently performative, with the user’s social network serving as an audience that observes what content users interact with and share. Motivations for engaging may or may not be linked to processing of campaign messaging. For example, it is possible that content that is highly engaging (eg, humorous or controversial content) does not drive behavioral change, that the desired behavioral change is not personally relevant to advocates who are keen to engage and promote the campaign (eg, ex-smokers), or that people do not engage (by liking, sharing, or commenting) with hard-hitting content that does drive behavioral change, as they may not want their peers to see their engagement with this type of content. Despite looking for indication of a relationship between engagement measures and priming step measures in this review, none of the included campaigns provided data that could allow for the analysis of correlations between these two types of measures. To understand whether engagement metrics are meaningful, future research studies need to specifically design campaign evaluations that look at whether people who undertake digital engagement actions are more or less likely to have changes in knowledge or attitudes, or even make the desired behavioral change [99]. It is only by gaining a greater understanding of the relationship of engagement measures with other evaluation measures that we know whether reporting engagement measures is at all meaningful [99,100].

### Measuring Media Platform Attribution

One of the major challenges facing practitioners is knowing where to invest resources given the diverse media landscape. The number of platforms is overwhelming, and without evidence of which are more useful at achieving campaign objectives, decisions are sometimes made based on opinions or trends. Therefore, this review examined whether campaign evaluations measured attribution, that is, how activity on each media platform used by the campaign contributed to the campaign’s outcomes. Despite this being important information, only a low proportion of campaigns (7/17) measured attribution. The methods used to measure attribution included survey self-report, using unique website tracking codes for different media format ads, and using an ecological study approach of correlating exposure of different media use combinations with reported campaign awareness and outcomes.

The majority of mass media campaigns use more than one media platform, as reflected in the campaigns included in this review. Previous research has shown that advertising campaigns on multiple platforms produces higher return-on-investment, and campaigns in sectors that are higher-involvement, such as pharmaceuticals, benefit most from synergistic campaigns using both traditional and digital media [101]. Therefore, while the trend toward multiplatform campaigns is clear, there is a great deal of uncertainty on how to accurately measure attribution in cross-platform marketing campaigns [102-104]. This is an even greater challenge in public health campaigns in comparison to marketing campaigns, as the final outcome to determine return-on-investment is not a purchase, but rather an attitudinal or behavioral change.

In all, 4 of the campaigns in this review used surveys or interviews to determine where people had encountered the campaign. However, this method has widely been found to be inaccurate, particularly where different media interact with one another or are viewed at the same time, making it difficult for people to recall where they encountered the campaign [105]. The study by Pettigrew et al [38] identified that people would often attribute their encounter with a campaign to television, even if this was unlikely to be the case. One campaign in this review (*SmokeFree Teen*) used unique website tracking codes on different media format ads to identify attribution. While this has the benefit of being objective, ad click-throughs underestimate the true impact of campaigns. Ad click-through rates have been steadily dropping over time to an average of 0.1% and have been shown not to have any relationship with ad effectiveness [86]. This may be because people instead search for the campaign on a search engine or manually type in a website address at a later time, rather than clicking on an ad at the time of viewing [24]. In addition, using ad click-throughs to measure attribution only captures the most recent encounter that an individual has with the campaign, not taking into account that earlier encounters with the campaign could have influenced their decision to click on the ad. Other methods of measuring attribution include passive systems of tracking exposure to campaigns, such as household meters to record when the TV is on or computer meters that monitor what websites are visited [106]. These methods are used by market research companies for population samples but were not used by any of the campaign evaluations in this review and are not widely used in public health campaigns as they are expensive to implement.

Given the absence of practical methods for campaign evaluators to accurately measure attribution for individual campaigns, there needs to be guidance provided to practitioners on what are generally the most effective combinations of media use. To develop such best practice guidelines, more studies examining the synergistic effects of different combinations of media platforms for public health mass media campaigns are required. The study design used by Allom et al [37] provides a good approach to developing a stronger understanding of the effectiveness of different combinations of media. By testing individual and combinations of media platform use at different times (such as TV only, TV and digital video, and web display and digital video) and then measuring campaign awareness and campaign-related events (website visits, calls to Quitline, registrations to quit program), the study provides an understanding of which combinations are more effective. This approach captures the synergistic effect of multiple media platforms, rather than attempting to simplify measurement to the first encounter with a campaign (eg, asking in a survey, “Where did you first see the campaign?”) or the last touchpoint with a campaign (eg, tracking click-throughs to a quit website). Further research building on this study would help generate evidence for best practice in cross-platform tobacco control campaigns. This could include replicating the study design with another campaign to validate findings and developing it further by asking about priming steps (eg, attitudes toward smoking) and/or trialing behaviors (eg, quit attempts) in addition to campaign awareness. Furthermore, future studies could explore the effect of varying the order of campaign exposure on different platforms, as it has been shown in advertising campaigns that TV first, then followed by digital, has a much larger synergistic effect than vice versa [101].

### Strengths and Limitations

One of the key strengths of this review is the use of peer-reviewed literature, marketing reports, grey literature, campaign websites, and social media sites to collect data for the campaigns. The triangulation of data provides a more comprehensive and practical view of how campaigns are currently evaluated.

This review included a wide range of campaigns in terms of scale, making comparison between campaigns difficult. However, the challenges in campaign evaluation identified in this review are common to all health-related campaigns, regardless of size and resourcing. The inclusion of English-only articles and the high representation of campaigns from English-speaking countries may limit the generalizability of this review’s findings and miss potential advances in non-English speaking countries. In addition, the large number of evaluation studies emanating from one campaign *(Tips from Former Smokers*) may also unevenly influence the findings of this review. The exclusion of campaigns about the use of e-cigarettes and waterpipe smoking is another limitation of this review, particularly as these forms of tobacco use are increasing in many populations, and campaigns in these areas may contain advances in the evaluation of digital media. Another limitation of this review is that a large proportion of articles were identified through hand-searching reference lists of included articles. This highlights the complexities in defining appropriate keywords for searching in this area and also supports the value of using this snowball method to ensure the majority of relevant literature is captured. Of note, specific social media–focused keywords were not included in the search strategy; however, many of the campaigns identified in this review use various social media platforms, suggesting that the overall approach has captured the main forms of social media use by mass media campaigns. In addition, future reviews could benefit from using PubMed searches to ensure newer journals not yet indexed by MEDLINE are included as well. The fragmented amount of information publicly available for some of the included campaigns is also a limitation of this review. Contacting organizations responsible for the campaign could provide more information; however, another review study found this method did not yield much additional information [107].

### Conclusions

This review examined how recent tobacco control campaigns that used traditional and digital media platforms were evaluated. It found that in today’s fragmented and rapidly evolving media environment, a wide and diverse range of measures and metrics were used in campaign evaluations, particularly for campaign activities relating to digital media use. Purposeful selection of metrics, and utilization of a conceptual framework can help practitioners and researchers make sense of the multitude of metrics and conduct evaluations that further our understanding of how best to use traditional and digital media to communicate health messages to target audiences.

## Appendix

Multimedia Appendix 1 Eligibility criteria for literature review.

Multimedia Appendix 2 Tobacco control campaigns including a digital media component and their evaluation methods.

## Abbreviations

GRPs: gross rating points
MEDLINE: Medical Literature Analysis and Retrieval System Online
TARPs: target audience rating points
