# Supplementary material for: Review of Evaluation Metrics Used in Digital and Traditional Tobacco Control Campaigns
Source: J Med Internet Res. 2020 Aug 11;22(8):e17432. doi: 10.2196/17432 (PMC7448186; doi:10.2196/17432)
Supplement: Multimedia Appendix 1 [file jmir_v22i8e17432_app1.pdf]

Stage 1a: Eligibility criteria for peer-review articles

Inclusion criteria

- Articles that included evaluation of a specific tobacco-related program that had a mass media campaign component. Campaigns were defined as purposive, public sector efforts to inform and influence a population or large segment of the population.

Exclusion criteria

- Did not reference a specific campaign by name.
- Article reported on recruitment of intervention participants using digital platforms.
- Intervention did not have a mass communication component.
- Experimental studies (not in real-world setting).
- Analyses of media or online coverage of tobacco messages.
- Formative research into message testing, or media use patterns to inform campaign design.
- Studies focused on validating scales by using a specific campaign as a case study.
- Studies that compared multiple campaigns, without evaluation of individual campaigns.

Stage 1b: Eligibility criteria for marketing reports

Inclusion criteria

- Reports that described tobacco control campaigns, regardless of whether evaluation results were included in the marketing report.
- Campaigns that included short period burst campaigns, or campaigns that drove audience to cessation services.
- Reports about individual initiatives that were part of a larger campaign.

Exclusion criteria

- Campaigns which were not specifically tobacco-related (e.g. heart health campaigns).
- Marketing campaigns by tobacco companies.
- Single public relations (PR) event campaigns and advocacy campaigns (e.g. introducing policy

about plain packaging) due to the different nature of intended outcomes.

Stage 2: Eligibility criteria for campaigns

Inclusion criteria

Campaign criteria

- Sufficient detail about how the campaign was conducted, including the media platforms employed, and how each platform was used.* Information about financial spend on each platform was useful, but not essential.
- Primary purpose of the intervention was campaign-related, with specific activities to promote a campaign message.
- Campaign included at least one digital component, with sufficient details of what the digital component involved.
- Campaign activities could be paid or unpaid.

Evaluation criteria

- Sufficient details on the methodology of how the evaluation was conducted.*

Exclusion criteria

- Insufficient information to assess the campaign activities, i.e. only general descriptions of campaign activities, such as ‘digital advertising’ or ‘social media campaigns’ with no further details provided.*
- Campaigns targeting e-cigarettes and waterpipe smoking.

*Information sourced from peer-reviewed journal articles or campaign reports (marketing literature or other grey literature). If available, additional information from websites or social media sites was used to help supplement this process.
