# Supplementary material for: Review of Evaluation Metrics Used in Digital and Traditional Tobacco Control Campaigns
Source: J Med Internet Res. 2020 Aug 11;22(8):e17432. doi: 10.2196/17432 (PMC7448186; doi:10.2196/17432)
Supplement: Multimedia Appendix 2 [file jmir_v22i8e17432_app2.pdf]

Table 4 - Tobacco control campaigns including a digital media component and their evaluation methods

CAMPAIGN	EVALUATION STUDY/ SOURCE	EVALUATION LEVEL	SUMMARY OF EVALUATION METHODS AND MEASURES
**16 Cancers (2015)** A state-wide campaign in Australia that ran for four months, raising awareness of the range of cancers associated with smoking by sharing emotional and graphic images and stories. The campaign used TV, digital platforms, radio, and out-of-home ads.	Allom et al., 2018 [37] Pettigrew et.al, 2018 [38]	*Awareness, Proximal Impact (Engagement) and Distal Impact Measures*	Cost-effectiveness time-series study assessing number of 'campaign events' (website visits, calls to telephone service, registrations to smoking cessation service or requests for QuitKit) for each burst of the campaign which used different media. Also measured campaign awareness for each campaign burst through a telephone survey.
**Be a Failure (2017)** A national campaign in Canada that ran for five months, encouraging smokers to understand that cessation often takes many quit attempts. The campaign used digital video, social media and out-of-home ads.	'Ministry of Health and Long-Term Care: Be a Failure' case study, 2018 [36]	*Process, Awareness, Proximal Impact (Engagement), Proximal Impact (Priming Steps) Measures*	Pre- and post-campaign market research survey asked smokers about campaign awareness, attitudes around quit attempts, intention to quit and whether they had sought out more information. Also used data from Google Analytics about traffic to campaign website. Campaign KPIs suggest process evaluation measures, but results not reported.
**Break it Off (2012)** A national campaign in Canada that ran for three months, encouraging young adult smokers to quit smoking by likening it with ending an unhealthy relationship. The campaign used a social marketing approach and was promoted using paid online and social media ads.	Baskerville, Azagba, Norman, McKeown & Brown, 2016 [60]	*Proximal Impact (Engagement), Proximal Impact (Priming Steps), Distal Impact and Outcome Measures*	Quasi-experimental study with an intervention and a comparison group (participants of a different intervention). Participants' intention to quit, actions towards quitting, and 7- and 30- day abstinence rates were measured pre- and post- campaign via questionaries. Study also reported website visits, installations of smartphone app and social media engagement metrics.

CAMPAIGN	EVALUATION STUDY/ SOURCE	EVALUATION LEVEL	SUMMARY OF EVALUATION METHODS AND MEASURES
**Fingerband campaign (2015)** A national campaign in Korea that targeted teenage smokers. The campaign used digital content, including a 'web-toon' and 'web-drama', as well as distributing physical campaign merchandise (wristbands with the campaign logo).	'Ministry of Health and Welfare: The Fingerband campaign' case study, 2016 [25]	*Process, Awareness, Proximal Impact (Engagement) and Outcome Measures*	The main evaluation measure of the campaign was teen population smoking rates over time. Also reported number of campaign participants, number of wristbands distributed and digital metrics such as campaign digital content views, comments, and review scores.
**Keep Trying (2013)** A state-wide campaign in Canada that ran for four weeks, targeting women aged 25-40 years who were smokers. The campaign used online, radio and OOH ads.	'Alberta Health Service: Tobacco Cessation – Keep trying' case study, 2015 [61]	*Proximal Impact (Engagement) and Distal Impact Measures*	Campaign evaluation consisted of measuring campaign website traffic, and registrations for cessation support services on the website.
**No judgements. Just help (2014)** A state-wide campaign in the USA that encouraged smokers to contact the redesigned smoking cessation service. The campaign used TV, radio, print, OOH and digital ads.	Keller et al., 2016 [72]	*Proximal Impact (Engagement), Distal Impact and Outcome Measures*	Observational study of smokers who utilised the quit service with data collected at registration and at a seven month follow up survey. Measures included quit attempts, and 30-day abstinence rates. The evaluation also used telephone service provider reports and Google Analytics to measure calls to cessation service, website visits and registrations to quit service.
	QUITPLAN Service: No judgements. Just help.' case study, 2015 [62]	*Proximal Impact (Engagement) and Distal Impact Measures*	Evaluation reported number of phone calls, web page views, quitting starter kit requests, with data from service provider and Google Analytics.

CAMPAIGN	EVALUATION STUDY/ SOURCE	EVALUATION LEVEL	SUMMARY OF EVALUATION METHODS AND MEASURES
**Personal Testimonies (Make Smoking History) (2012)**<br>A state-based campaign in Australia that ran for two months, targeting 25-39 year old male smokers by sharing the personal testimonies of two smokers. The campaign used print, radio and online ads.	Clayforth et al., 2014 [59]	*Proximal Impact (Engagement) and Distal Impact Measures*	Cost-effectiveness time-series study assessing number of campaign responses (calls to telephone service, accessing specific web address provided, web searches to locate the website and registrations to smoking cessation service) for each burst of the campaign which used different media.
**Quit the Denial (2013)**<br>A state-based campaign in Canada targeting young adults, using humour to challenge the social norm of acceptability of 'social smoking'. The campaign was run predominately online using video and display ads, in additional to out-of-home promotions.	Ontario Ministry of Health and Long-Term Care: Social smoking campaign' case study, 2014 [26]	*Process, Awareness, Proximal Impact (Engagement), and Proximal Impact (Priming Steps) Measures*	Evaluation reported process measures of video views, "earned impressions", and number of "social smokers" reached. Also reported engagement measures of proportion of people talking about 'social smoking' online and in social media, proportion who sought further information, and priming step measures of knowledge and attitudes (but unclear how this data was obtained).
**SmokeFree Teen (2013)**<br>A national campaign in the USA that ran for almost three months, aimed at encouraging adolescent smokers to access smoking cessation resources. The campaign used TV, radio, online and social media ads.	Sanders et al., 2018 [8]	*Process, Awareness, Proximal Impact (Engagement), and Distal Impact Measures*	Evaluation using digital metrics to collect data on exposure to digital ads, clicks on campaign ads, and campaign outcomes (visits to campaign website, sign-ups to SMS program, smartphone app downloads, Facebook fans and Twitter followers).

CAMPAIGN	EVALUATION STUDY/ SOURCE	EVALUATION LEVEL	SUMMARY OF EVALUATION METHODS AND MEASURES
**Stop before the suffering starts (Breathless & Symptoms) (2013)** A national campaign in Australia that ran for three months to encourage quit attempts by highlighting the pain associated with smoking-related illnesses. The campaign used TV, print, out-of-home and online video ads.	Myers & Blackmore, 2013 [14] and von Weiler, Bayard & Sheard, 2014 [39]	*Process, Awareness, Proximal Impact (Engagement), Proximal Impact (Priming Steps) and Distal Impact Measures*	Process measures of TARPS reported for television ads. Telephone interviews were conducted to measure campaign awareness, channel attribution, campaign response, beliefs about health harms of smoking, attitudes towards smoking, intention to quit, actions towards a quit attempt and actual quit attempts.
**Stoptober (2012 – present)** A national campaign that has been run annually in the UK every October. The campaign aims to create a social movement to encourage people to quit smoking. It is a social marketing campaign that uses TV, print, radio, online and social media promotions.	Brown et al., 2014 [74]	*Distal Impact Measures*	Monthly nationally representative household surveys conducted in the years prior to the campaign, and in the first year of the campaign, measuring past-month quit attempt rates.
	Arden, Buckley, Hirst, Shardlow & Walmsley, 2016 [67]	*Proximal Impact (Engagement), Proximal Impact (Priming Steps), Distal Impact and Outcome Measures*	Using Public Health England's Tobacco Simulation Model, the evaluation estimated number of quit attempts, successful quit attempts (greater than 4 weeks) in the population, proportion who used the campaign support tools, and proportion who believed lots of people were quitting together. The evaluation also reported cigarette sale volumes, internet search term volumes, and 'social mentions'.
	Public Health England: Stoptober 2016 Facebook Messenger Bot' case study, 2017 [69]	*Proximal Impact (Engagement)*	Evaluation reported number of people using the campaign Facebook Messenger Bot, and engagement with the email communications.
	Public Health England, 2017 [75]	*Awareness, Proximal Impact (Engagement), Distal Impact and Outcome Measures*	Online interviews with current and recent ex-smokers to measure brand awareness, quit attempts and sustained quit attempts. Digital metrics were collected to identify uptake of the Facebook Chatbot

CAMPAIGN	EVALUATION STUDY/ SOURCE	EVALUATION LEVEL	SUMMARY OF EVALUATION METHODS AND MEASURES
	Public Health England, 2018 [63]	*Awareness, Proximal Impact (Engagement), Distal Impact and Outcome Measures*	Using marketing and advertising tracking surveys, the evaluation collected measures of brand awareness, response to campaign message quit attempts and sustained quit attempts<br>It also used digital metrics to measure campaign-related online searches, website visits, downloads of app, sign-ups to eCRM programme and uptake of Facebook Chatbot.
**Take it right outside (2014)**<br>A national campaign in Scotland that ran for four months, aiming to educate smokers who are parents on the dangers of secondhand smoke for their children. The campaign used TV, radio, out-of-home and online ads.	'Scottish Government: Second-hand Smoke – Take it right outside' case study, 2014 [70]	*Proximal Impact (Priming Steps)*	Survey to measure knowledge of effect of secondhand smoke on children, and attitudes about whether it is acceptable to smoke around children.
	Rowa-Dewar & Amos, 2016 [55]	*Awareness and Outcome Measures*	Pre- and post-campaign semi-structured interviews with parents measuring campaign awareness, message response, and behavioural change.
	Progressive, 2014 [40]	*Awareness, Proximal Impact (Engagement), Proximal Impact (Priming Steps), Distal Impact Measures*	Face-to-face interviews, with optional self-complete questionnaire to measure campaign awareness (for each media channel used), actions taken as a result of the ad, smoking behaviours around children, attitudes about smoking and perceived risk of secondhand smoke to children.
**The Facts Now (2015)**<br>A state-wide campaign in the USA targeting teens and young adults. The campaign was primarily online, using shareable content, but also utilised events, TV and radio promotions.	Tobacco Free Florida: Auctioneer' case study, 2016 [27]	*Proximal Impact (Engagement) and Outcome Measures*	The evaluation reported website visits and engagements, Twitter followers and engagements, Facebook fans and engagements and YouTube views. It also cited the population teen smoking rate.
**The Real Cost (2014 – present)**<br>A multi-year national campaign in the USA aimed at preventing youth (12-17 year olds)	Duke et al., 2015 [15]	*Awareness Measures*	Longitudinal in-person and online survey with target audience measuring campaign awareness, brand awareness and perceived effectiveness of the campaign message.

CAMPAIGN	EVALUATION STUDY/ SOURCE	EVALUATION LEVEL	SUMMARY OF EVALUATION METHODS AND MEASURES
from becoming smokers. The campaign used TV, radio, print, out-of-home digital and social media promotions.	Farrelly et al., 2017 [41]	*Awareness and Outcome Measures*	Cohort study of online or in-person interviews pre- and post-campaign, collecting data on self-reported campaign exposure, frequency of exposure, and smoking initiation.
	Huang et al., 2017 [42]	*Awareness and Proximal Impact (Priming Steps) Measures*	Telephone survey measuring campaign awareness, attitudes about tobacco products, and risk perceptions of smoking.
	Kranzler, Gibson & Hornik, 2017 [43]	*Awareness and Proximal Impact (Priming Steps) Measures*	Observational study using telephone survey to measure recall of campaign ad, anti-smoking beliefs targeted by the campaign and intention to smoke.
	Chew, Kim, Chen, Ruddle & Morgan-Lopez, 2018 [77]	*Process Measures*	Social network analysis of Twitter accounts that helped maximise the reach of the campaign message.
	MacMonegle et al., 2018 [78]	*Cost-effectiveness evaluation*	Cost-effectiveness evaluation of campaign based on cost per quality-life adjusted year saved, and monetary return on investment.
	Food and Drug Administration: Little Lungs' case study, 2017 [29]	*Process and Proximal Impact (Engagement) Measures*	Evaluation of the online stop-animation video series reported number of views on YouTube, Facebook and Instagram, and number and rate of 'social engagements'.
	Duke et al., 2018 [16]	*Awareness and Proximal Impact (Priming Steps) Measures*	Longitudinal study with a baseline survey and two post-campaign follow-up surveys. Measured tobacco-related beliefs (both related to campaign, and not related to campaign). Exposure to campaign measured by self-report and based on market-level TARPs.

CAMPAIGN	EVALUATION STUDY/ SOURCE	EVALUATION LEVEL	SUMMARY OF EVALUATION METHODS AND MEASURES
**The Smoking Kid (2012)** A national campaign in Thailand that used a single video on social media to encourage introspection among smokers to motivate them to quit smoking.	THPF: The Smoking Kid – A personal message to smokers' case study, 2013 [28]; and 'Thai Health Promotion Foundation: Smoking kid' case study, 2015 [73]	*Process and Distal Impact Measures*	Evaluation reported number of YouTube video views, earned media value and number of calls to smoking cessation service.
**Tips from Former Smokers (2012 - present)** A multiyear national campaign in the USA that shares testimonies from people who live with tobacco-related diseases. The campaign uses TV, radio, out-of-home, digital video, digital display, search and social media ads.	Augustson et al., 2012 [65]	*Proximal Impact (Engagement) and Distal Impact Measures*	Analysis of smoking cessation phone service call volume and website visits data before, during and immediately after campaign period (using data from service provider and web metrics).
	McAfee, Davis, Alexander, Pechacek & Bunnell, 2013 [44]	*Awareness, Distal Impact and Outcome Measures*	Cohort study of smokers and non-smokers with baseline and follow-up surveys measuring quit attempts, sustained quit attempts, cessation recommendation to friends/family over previous three months and campaign awareness.
	Bright et al., 2013 [66]	*Proximal Impact (Engagement) and Distal Impact Measures*	Analysis of smoking cessation phone service call volume and website visits before, during and after campaign (using data from service provider and web metrics). Evaluation examined the effect of 'pulsing' the national television ads whilst local television and online ads ran continuously.
	Duke, Hansen, Kim, Curry & Allen, 2014 [79]	*Process Measures*	Descriptive overview of how state tobacco control programs used and disseminated campaign content on social media (Facebook, Twitter and YouTube).
	Emery, Szczypka, Abril, Kim & Vera, 2014 [56]	*Awareness Measures*	Assessed Twitter content related to campaign for relevance, message content, and 'fear' appeal characteristics.

CAMPAIGN	EVALUATION STUDY/ SOURCE	EVALUATION LEVEL	SUMMARY OF EVALUATION METHODS AND MEASURES
	Zhang, Vickerman, Malarcher & Mowery, 2014 [17]	*Distal Impact and Outcome Measures*	Measured cessation outcomes (24hr quit attempt and 7+ days abstinence) of people who used Quitline phone services, and analysed in relation to campaign exposure (based on GRPs of area code).
	Ayers, Althouse & Emery, 2015 [68]	*Proximal Impact (Engagement)*	Measured Google searches for campaign-related keywords (e.g. amputation, asthma and smoking) and cessation-related searches before and during campaign.
	Chung, 2015 [30]	*Process, Awareness and Proximal Impact (Engagement) Measures*	Quantitative (number of views and comments) and qualitative (sentiment and content analysis) evaluation of campaign's YouTube videos.
	Davis et al., 2015 [18]	*Distal Impact Measures*	Analysed call volume to Quitline relative to the weekly media market level campaign GRPs for television and radio.
	Duke et al., 2015 [45]	*Awareness and Proximal Impact (Priming Step) Measures*	Longitudinal online survey examining relationship between exposure to the campaign and changes in beliefs, tobacco related cognitions and intentions to quit smoking.
	Huang et al., 2015 [46]	*Awareness, Proximal Impact (Priming Step) and Distal Impact Measures*	Pre- and post-campaign cohort study, assessing knowledge of smoking risks, awareness and use of cessation resources and quitting behaviours in relation to exposure to campaign ads.
	Komfield, Smith, Szczypka, Vera & Emery, 2015 [5]	*Process and Proximal Impact (Engagement) Measures*	Sampling of online media sites to identify coverage of campaign. Identified content was coded for content, inclusion of multimedia and measures of audience engagement.

CAMPAIGN	EVALUATION STUDY/ SOURCE	EVALUATION LEVEL	SUMMARY OF EVALUATION METHODS AND MEASURES
	Xu et al., 2015 [80]	*Cost-effectiveness evaluation*	Cost-effectiveness evaluation based on cost per successful quit, cost per premature death averted, cost per life year saved, and cost per quality-adjusted life year gained. Behavioural outcomes measures based on McAfee et al., 2013 study.
	Zhang, Vickerman, Malarcher & Carpenter, 2015 [81]	*Process and Awareness Measures*	Examined caller characteristics to smoking cessation phone service during campaign period compared with a similar period in the previous year. Also examined how callers had heard about the quit service (e.g. television media, other media, referral)
	Chung, 2016 [82]	*Process Measures*	Identified parties who tweeted about the campaign on Twitter, and who played central roles in disseminating health campaign messages.
	Davis et al., 2016 [19]	*Process and Awareness Measures*	Assessed the effect of variation in dose of digital video and television ads on awareness of campaign through setting up different doses in different media markets. Measured self-reported exposure to campaign, media format they recall seeing campaign and frequency.
	Kim et al., 2016 [24]	*Process and Proximal Impact (Engagement) Measures*	Collected data from a web panel tracking measuring visits to campaign sites and other related smoking cessation websites, and search queries using related keywords.
	Neff et al., 2016 [47]	*Awareness, Proximal Impact (Priming Step), Distal Impact and Outcome Measures*	Evaluated phase 2 of the 2014 campaign using pre- and post-campaign online surveys in a nationally representative longitudinal cohort. Measures included self-reported campaign recall, quit attempts, intention to quit and successful quit attempts.
	Shafer et al., 2016 [20]	*Process and Proximal Impact (Engagement) Measures*	Analysed relationship between geographical and temporal variations in dose of television and digital video campaign ads with visits to campaign website.
	Zhang et al., 2016 [83]	*Distal Impact Measures*	Using call volume data, the study examined the effect of campaign ads on calls to smoking cessation phone services of states with and without alternative phone numbers.

CAMPAIGN	EVALUATION STUDY/ SOURCE	EVALUATION LEVEL	SUMMARY OF EVALUATION METHODS AND MEASURES
	Zhao & Cai 2016a [49]	*Awareness Measures*	Assessed impact of campaign on adolescents, using National Youth Tobacco Survey (a cross sectional survey of school students). The study measured ad recall (promoted by description) and smoking status.
	Zhao & Cai 2016b [48]	*Awareness and Proximal Impact (Priming Step) Measures*	As above (Zhao, 2016a), but also analysed exposure to campaign with intention to quit and smoking susceptibility.
	Abril, Szczypka & Emery 2017 [57]	*Awareness/ Proximal Impact (Engagement) Measures*	Analysed campaign-related tweets for fear control responses.
	Davis et al., 2017 [58]	*Awareness and Distal Impact Measures*	Examined whether perceived effectiveness of ads was associated with quit attempts using survey data from nationally representative longitudinal cohort study of smokers at baseline and follow up. Measures of perceived effectiveness (PE) were rated after viewing ad. PE measures included whether 'ad worth remembering', 'grabbed my attention', powerful, informative, meaningful, or convincing.
	England et al., 2017 [76]	*Outcome Measures*	Analysed effect of campaign on smoking cessation by pregnant women. Exposure to campaign was measured based on campaign air dates, and smoking status was ascertained from birth certificates.
	McAfee et al., 2017 [50]	*Awareness, Proximal Impact (Priming Step), Distal Impact and Outcome Measures*	Study measured the effect of increasing doses of television campaign ads. Nationally representative survey measured awareness of campaign, knowledge of smoking-related diseases, quit attempts, intention to quit smoking; and for non-smokers: communication with friends or family about smoking dangers.

CAMPAIGN	EVALUATION STUDY/ SOURCE	EVALUATION LEVEL	SUMMARY OF EVALUATION METHODS AND MEASURES
	Davis et al., 2018 [21]	*Process, Proximal Impact (Priming Step) and Distal Impact Measures*	Analysed seven waves of nationally representative surveys (baseline and six follow-ups) from 2012-2015. Measured quit attempts and intention to quit, and compared against television ad GRPs.
	Murphy-Hoefer et al., 2018 [84]	*Distal Impact and Outcome Measures*	Used data from Davis et al. [21] and Neff et al. [47] to estimate population number of campaign-attributable quit attempts and sustained quit attempts from 2012-2015.
	Zhang et al., 2018 [22]	*Process and Distal Impact Measures*	Examined effect of campaign on Spanish smoking cessation phone services use by analysing number of calls to Spanish Quitline and exposure to campaign television ads.
**Truth FinishIt (2014 - present)** A national campaign in the USA targeting youth (15-21 year olds), with the aim of changing the social norm of smoking. The campaign uses TV, digital display ads, online video and social media ads.	Evans et al., 2016 [51]	*Awareness and Proximal Impact (Priming Step) Measures*	Partly a feasibility study to develop a 'brand equity' scale for this phase of the campaign. An online survey was used to collect data on campaign exposure, and attitudes and beliefs about tobacco use.
	Vallone et al., 2016 [52]	*Awareness, Proximal Impact (Engagement) and Proximal Impact (Priming Step) Measures*	Evaluation used data from a marketing survey on brand awareness and anti-tobacco attitudes; and data from longitudinal cohort interviews measuring campaign awareness and anti-tobacco industry attitudes. Social media engagement data (engagement on Facebook, Twitter, YouTube and Instagram) was also collected using a third party site
	'truth: ''Finish It"' case study, 2016 [31]	*Outcome Measures*	Evaluation reported population smoking rates over time.
	'Truth Initiative: Left Swipe Dat' case study, 2016 [64], and 'truth: "Left swipe dat"' case study, 2016 [71]	*Process and Proximal Impact (Engagement) Measures*	Evaluation of the 'Left Swipe Dat' video reported earned media impressions, campaign website views and video views. The report also included changes in brand equity ratings and changes in attitudes about people who smoke.

CAMPAIGN	EVALUATION STUDY/ SOURCE	EVALUATION LEVEL	SUMMARY OF EVALUATION METHODS AND MEASURES
	Hair et al., 2017 [35]	*Process, Awareness and Proximal Impact (Engagement) Measures*	Evaluation used cross-sectional online surveys and digital metrics to measure awareness of campaign ads which aired during popular television events, and level of social media engagement. The evaluation also reported on GRPs and digital impressions.
	Vallone et al., 2017 [53]	*Awareness, Proximal Impact (Priming Steps) and Outcome Measures*	Using the campaign longitudinal cohort study (interviews at baseline, and every six months), evaluation collected data on brand equity, smoking status, intention to quit smoking, anti-tobacco attitudes and ad awareness.
	Evans et al., 2018 [54]	*Awareness, Proximal Impact (Priming Steps) and Outcome Measures*	Using the campaign longitudinal cohort study, evaluation analysed measures of campaign awareness, brand equity, campaign-related attitudes, anti-tobacco sentiment and current smoking status.
	Vallone et al., 2018 [23]	*Awareness and Proximal Impact (Priming Steps) Measures*	Using the campaign longitudinal cohort study, evaluation analysed measures of campaign awareness (including dose), campaign-related attitudes, anti-tobacco sentiment and intention to smoke.
	Weir et al., 2018 [85]	*Cost-effectiveness evaluation*	Cost-effectiveness evaluation of campaign based on expenditure, estimated lifetime treatment costs saved and QALYs saved
	'Truth Initiative: #StopProfiling' case study, 2018 [32]	*Process and Proximal Impact (Engagement) Measures*	Evaluation of the #StopProfiling campaign push reported number of video views, social engagements, website traffic, sign-ups to campaign and earned media impressions.
